# The use of driving endonuclease genes to suppress mosquito vectors of malaria in temporally variable environments

**DOI:** 10.1186/s12936-018-2259-8

**Published:** 2018-04-04

**Authors:** Ben Lambert, Ace North, Austin Burt, H. Charles J. Godfray

**Affiliations:** 1 Department of Zoology, University of Oxford, South Parks Road, Oxford, OX1 3PS UK; 2 Department of Infectious Disease Epidemiology, School of Public Health, Faculty of Medicine, Imperial College London, St. Mary’s Campus, Norfolk Place, London, W2 1PG UK; 3 Department of Life Sciences, Imperial College London, Silwood Park, Ascot, Berks, SL5 7PY UK

**Keywords:** *Anopheles*, Rainfall, Homing endonucleases, Epidemiology, Vector control, Climate-modelling, Spatial modelling, Weather

## Abstract

**Background:**

The use of gene drive systems to manipulate populations of malaria vectors is currently being investigated as a method of malaria control. One potential system uses driving endonuclease genes (DEGs) to spread genes that impose a genetic load. Previously, models have shown that the introduction of DEG-bearing mosquitoes could suppress or even extinguish vector populations in spatially-heterogeneous environments which were constant over time. In this study, a stochastic spatially-explicit model of mosquito ecology is combined with a rainfall model which enables the generation of a variety of daily precipitation patterns. The model is then used to investigate how releases of a DEG that cause a bias in population sex ratios towards males are affected by seasonal or random rainfall patterns. The parameters of the rainfall model are then fitted using data from Bamako, Mali, and Mbita, Kenya, to evaluate release strategies in similar climatic conditions.

**Results:**

In landscapes with abundant resources and large mosquito populations the spread of a DEG is reliable, irrespective of variability in rainfall. This study thus focuses mainly on landscapes with low density mosquito populations where the spread of a DEG may be sensitive to variation in rainfall. It is found that an introduced DEG will spread into its target population more reliably in wet conditions, yet an established DEG will have more impact in dry conditions. In strongly seasonal environments, it is thus preferable to release DEGs at the onset of a wet season to maximize their spread before the following dry season. If the variability in rainfall has a substantial random component, there is a net increase in the probability that a DEG release will lead to population extinction, due to the increased impact of a DEG which manages to establish in these conditions. For Bamako, where annual rainfall patterns are characterized by a long dry season, it is optimal to release a DEG at the start of the wet season, where the population is growing fastest. By contrast release timing is of lower importance for the less seasonal Mbita.

**Conclusion:**

This analysis suggests that DEG based methods of malaria vector control can be effective in a wide range of climates. In environments with substantial temporal variation in rainfall, careful timing of releases which accounts for the temporal variation in population density can substantially improve the probability of mosquito suppression or extinction.

**Electronic supplementary material:**

The online version of this article (10.1186/s12936-018-2259-8) contains supplementary material, which is available to authorized users.

## Background

Vector control lies at the forefront of efforts to combat malaria, a situation that is expected to continue in the foreseeable future [1, 2]. Insecticides, delivered via bed nets and indoor residual spraying, are the current mainstay of vector control and their widespread application has helped bring about dramatic reductions in malaria incidence over recent years [1]. Insecticide-based programmes alone, however, are not expected to be sufficient to eliminate malaria from the worst affected parts of sub-Saharan Africa, where climatic and socio-economic conditions are particularly conducive to endemic transmission [3, 4]. Moreover, the emergence of insecticide resistance in vector populations threatens the continuing efficacy of these measures [5]. Thus, there is considerable need for innovative complimentary methods of vector control [6].

Genetic manipulation using driving endonuclease genes (DEGs) offers the potential of suppressing vector populations [7]. DEGs spread rapidly in populations where they are introduced by using a process known as ‘homing’, where heterozygous cells are converted to homozygotes [8]. For use in vector control, a DEG could be inserted into a functional mosquito gene in order to induce a population-wide knockout of that gene [9, 10]. Alternatively, a DEG could be inserted onto the Y-chromosome so that in heterozygote males it disrupts the X-chromosome and thus biases the sex-ratio towards male offspring (“Y-drive”). Both approaches aim to either suppress or eliminate the target vector population.

The potential of these approaches has been demonstrated in the laboratory [9, 11–13], and investigated using mathematical models [7, 14–18]. The earlier models considered DEG releases in large panmictic populations to help understand the population load a DEG could impose in idealized conditions [7], how this depends on the specific nature of the DEG deployed [14], and the implications for malaria reduction [15]. More recently, North et al. [16] investigated how the spread of a DEG may be affected by the spatial structure of natural populations in heterogeneous landscapes. This work suggests that spatial variation in habitat quality does not affect the likelihood of spread in landscapes where the resources required by mosquitoes are abundant. In these landscapes DEG establishment and spread, therefore, occurs deterministically (i.e. the outcome is relatively certain and not affected by random events). However, in landscapes where these resources are distributed more sparsely, a DEG may become stochastically extinct before reaching all parts of the population. Real landscapes are likely a patchwork of areas with abundant mosquito resources, and those where resources are distributed more sparsely. Areas with sparse resources may affect the performance of a released DEG by providing barriers to its wider spread. Temporal variation in rainfall may be important because members of the *Anopheles gambiae* complex, which include the most important malaria vectors in Africa, tend to develop as larvae in small temporary water bodies created by rain [19–21]. The population dynamics of these species are, therefore, to varying degrees, sensitive to fluctuations in rainfall [20–22].

The aim of this paper is to investigate how fluctuations in rainfall will influence the performance of a Y-drive DEG in environments with sparse resources for mosquitoes and thus low-density populations, where the spread of a DEG is most sensitive to the release protocol [16]. Here a flexible model of daily precipitation is introduced which enables the generation of artificial rainfall data with specified characteristics [23]. This is combined with the mosquito population model of North et al. [16] to explore DEG releases in different climates. In particular, the roles of seasonality and random fluctuations in rainfall are explored and also how these forms of variability should be accounted for when planning releases. Two case-studies, Bamako in Mali and Mbita in Kenya, are used to investigate the combined influence of seasonal and random variability in these locations. These locations were chosen because they exemplify two distinct climatic regions of Africa where malaria incidence is high and DEGs may eventually be deployed (the Sahel and tropical east Africa, respectively), and in each case historical data is used to fit the rainfall model. In summary, this analysis addresses the following questions. (1) When is the best time to release a DEG in a strongly seasonal environment? (2) How will shorter-term random variability in rainfall affect the performance of a DEG? (3) What is the optimal release strategy in the two case-study locations?

## Methods

### Mosquito population model

The model used extends the spatially explicit and stochastic model of *Anopheles* population dynamics developed by North et al. [16], by incorporating temporal variability in environmental conditions. Since the demographic component of both the current model and its predecessor are the same, only a brief review of the assumptions is provided here. The model considers a population of mosquitoes that are each characterized by their sex, life-stage (juvenile or adult), genotype (whether or not they possess DEGs), and location in two-dimensional space. The landscape consists of small water bodies (‘breeding sites’), and houses (‘feeding sites’). Density dependence occurs only in the juvenile stage due to larval competition for food and is modelled by an increase in the rate of juvenile mortality. The length of the juvenile stage is assumed fixed at 9 days. Whilst it has been shown that temperature modulates the rate of development of the larval and pupal stages [24], here the focus is on the effects of rainfall and, hence, temperature is not included temperature in the model.

Emergent adult females go through a series of gonotrophic cycles, and are categorized by whether they are searching for males if they have not yet mated, `feeding sites’ in order to blood-feed, or `breeding sites’, in which to oviposit. The duration of the gonotrophic cycle has long been known to be sensitive to temperature [25] and future work may include this effect. Adult female mosquitoes move through the landscape at rates governed by their proximity to the objects they are seeking giving rise to area-restricted search behaviour. Following North et al. [16], it is assumed that adult males do not disperse, and are confined to the vicinity of the breeding sites.

In the simulations, the males are categorized by whether their Y-chromosome carries the DEG, and mated females by whether the sperm they carry is wild-type or DEG. It is assumed that the DEG acts in males to bias the fraction of Y-bearing gametes but that there is no effect on sperm number and hence female fertility. Further, it is assumed that 85% of the X-chromosomes are shredded so a female mated with a DEG bearing male will produce ~ 87% male offspring.

### Linking rainfall with breeding site density

This mosquito model was previously used to explore how landscape structure will influence the deployment of DEGs, by simulating releases across a wide range of feeding and breeding site distributions [16]. The analysis considered only static feeding site distributions, although the breeding sites were allowed to dynamically appear and disappear to reflect the fact that *Anopheles* larvae often develop in small water bodies subject to rainfall and evaporation [19–21]. North et al. [16] assumed that the rates of site creation and destruction were unchanging and equal in any given landscape, so that the average number of sites per unit area remained constant through time.

To investigate the role of climate and weather, it is supposed that variation in rainfall influences the rate of creation of breeding sites. The rainfall amount on day d is denoted by *θ*_*d*_ and so {*θ*_*d*_} is the time-series of daily rainfall. The population model is linked to weather by supposing that on a given day, *d*, breeding sites are randomly created at a rate *χ* × *κ* × *θ*_*d*_ per unit area, which means that the number of sites created on day *d* is Poisson distributed with expectation also equal to *χ* × *κ* × *θ*_*d*_ per unit area. Sites are destroyed at rate *χ* per site, which means the longevity of a given site is exponentially distributed with mean 1/*χ*. Note that a large value of *χ* means breeding sites appear quickly during rain and disappear quickly during drought, and thus this parameter is used to control the lag between rainfall and breeding site density. The parameter *κ* will be used to control the long-run average density of breeding sites, allowing us to investigate DEG releases across landscapes that share similar climates yet differ in their overall extent of breeding habitat.

The authors are aware of only a small number of field studies that have reported breeding site densities [19, 26–28], all of which were based in Western Kenya. Despite being from the same region, these studies report a wide range of densities, from ≈ 13 km^−2^ [26] to a rainy season peak of ≈ 330 km^−2^ [19], reflecting different local geographies. In this paper, average breeding site densities in the range 100–240 km^−2^ are considered. House density, like breeding site density, is a parameter that varies greatly across different landscapes; here the simulations are restricted to “sparse” landscapes by setting house density at a low level of 4 km^−2^.

### A general model for rainfall

The rainfall dynamics are modelled using the procedure first described by Bárdossy and Plate [23]. A time-series {*W*_*d*_} is considered that represents an aggregate measure of the various meteorological processes influencing rainfall, which is (subsequently) transformed to yield the rainfall time-series {*θ*_*d*_}. Rather than attempting to model the meteorological processes explicitly, it is assumed that on each day, *d*, *W*_*d*_ is a random variable that depends on three factors, (i) the conditions on the previous day (*W*_*d*−1_), (ii) the *trend* in meteorological conditions ({*μ*_*d*_}), and (iii) random noise ($\varepsilon_{d} \sim N\left( {0,\sigma^{2} } \right)$). Specifically, {*W*_*d*_} is a first-order autoregressive (AR-1) process given by the equation,

1 $$W_{d} = \rho W_{d - 1} + \left( {1 - \rho } \right)\mu_{d} + \varepsilon_{d}$$

where the parameter *ρ* controls the degree to which conditions are dictated by recent history rather than the long term trend in weather conditions, {*μ*_*d*_}, which may incorporate a seasonal cycle. The variance of the noise term *σ*^2^ is used to control the extent of randomness. Since the time-series {*W*_*d*_} may shift between positive and negative values, it is necessary to transform the series to derive the rainfall time-series {*θ*_*d*_}. Following Bárdossy and Plate [23], the following transformation is used,

2 $$\theta_{d} = \left\{ \begin{array}{ll} W_{d}^{\beta } &\quad {\text{for}}\;\;W_{d} > 0 \\ 0 & \quad {\text{for}} \;\;W_{d} \leq 0 \\ \end{array} \right.$$

where the exponent β is used to control the variance in the rainfall distribution at any particular location.

The flexibility of this model lies in the ability to manipulate the autocorrelation, noise, and trend of the simulated rainfall series. In the analyses that follow, first two limiting cases are explored before fitting the model using data from Bamako in Mali and Mbita in Kenya. In the first limiting case, rainfall is described by a sinusoidal trend with annual periodicity $\left( \mu_{d} = \mu_{0} { \sin }\left( {\frac{2\pi d}{365}} \right)\right)$ and no noise (*σ* = 0). In the second, rainfall is subject to noisy and possibly autocorrelated variability (*σ* > 0, *ρ* ≥ 0) yet with no underlying trend (*μ*_*d*_ = *μ* = constant).

**Fig. 1:**
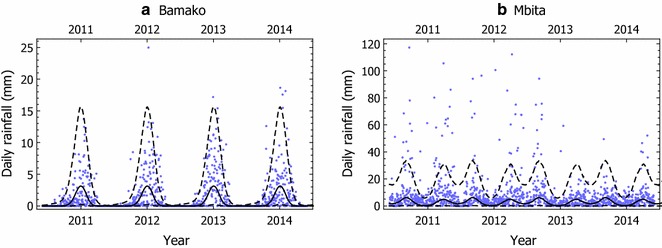
Actual rainfall (blue dots) versus quantiles of simulated (black lines) series for Bamako, Mali and Mbita, Kenya. In each panel, the solid line and two dashed lines represent the median, 5%, and 95% quantiles across 500 simulations of the daily rainfall series, which have been smoothed using 30-day moving averages

The model was fitted to the two case-study locations using 20 years of daily precipitation data in each case (1st January 1995 to 31st December 2014, obtained from the “ERA-interim reanalysis”, which is available from the European Centre for Medium-Range Weather Forecasts [29]). The parameter *β* was fixed a priori to give a good correspondence between simulated and actual rainfall series (Table 1, and Fig. 1, *β* = 4 for Bamako and *β* = 5 for Mbita). These values of *β* were used to compute {*W*_*d*_} by transforming the rainfall series (Eq. 2), where it was assumed that *W*_*d*_ = 0 on days with no rainfall. To estimate the trend {*μ*_*d*_}, the mean value of *W*_*d*_ was computed for each day of the year, averaged over the 20 year period. The resulting series {*μ*_*d*_}, was then approximated by a Fourier series with *K* harmonics,

**Table 1:** A comparison between rainfall data and model simulated data for Bamako, Mali, and Mbita, Kenya

Characteristic	Bamako		Mbita	
	Observed	Simulated	Observed	Simulated
Mean wet day amount (mm)	4.90	4.65	9.13	9.76
Standard deviation of wet day amount (mm)	4.80	4.61	13.30	13.90
Median amount (mm)	0.00	0.04	3.35	1.82
Prob. (dry tomorrow|dry today)	0.87	0.90	0.65	0.68
Prob. (wet tomorrow|wet today)	0.67	0.66	0.85	0.79
Prob. (wet)	0.28	0.23	0.70	0.60
Mean wet spell length (days)	7.65	9.58	2.87	3.10
Mean dry spell length (days)	2.99	2.91	6.61	4.68
Standard deviation of monthly totals (mm)	56.78	50.88	103.14	102.47
Maximum daily precipitation (mm)	110.75	42.26	158.95	222.83
Lag 1 autocorrelation	0.41	0.46	0.29	0.51

For simulated series, the values represent averages across 100 replicates

3 $$\mu_{d} = \mathop \sum \limits_{k = 0}^{K - 1} a_{k} \cos \left( {\frac{2\pi dk}{365}} \right).$$

*K* = 4 was chosen, which generated smoothed approximations of the {*μ*_*d*_} series, and produced rainfall series that closely approximated the actual data (Table 1 and Fig. 1). Finally, *ρ* and *σ* were estimated by maximum likelihood.

### DEG releases

The simulation environment was a square domain of area 16 km^2^ with periodic boundary conditions. In all cases simulations were run for a period of 50 days before the introduction of mosquitoes, in order to allow the breeding site dynamics to reach equilibrium. Similarly, for all replicates the wild-type population was run for a period of at least 2 years before the DEGs were introduced, again in order to allow the population to reach equilibrium. DEGs were introduced by distributing a number of males at random across all the breeding sites in the landscape. The default release number was 500 males, though this number was varied to assess the sensitivity of the results to this parameter. After the introduction, the simulations were allowed to run for a further 6 years, to allow sufficient time for a DEG to act.

For the purely deterministic sinusoidal model, the releases occurred at four equally-spaced times of the year, corresponding to the start of the wet season, the middle of the wet, the start of the dry season, and the middle of the dry season. For the model with short-term variation in rainfall around a constant mean, all releases occurred exactly 2 years after the simulations began. Finally, for the models using rainfall fit to data from Bamako, Mali and Mbita, Kenya, releases in each month of the year were simulated (the actual release occurring mid-month).

## Results

### DEG dynamics in a constant environment

In a constant environment, a DEG will either spread to fixation or fail to establish [16]. If fixation occurs, the load will either suppress or drive to extinction the population, depending largely on the intrinsic growth rate of the target mosquito population [15]. Consider, for example, a release site where unvarying rainfall results in a constant breeding site density of 142 km^−2^. If the density of houses was assumed to be greater than 8 km^−2^, fixation occurred in 100 out of 100 simulations. In a sparser landscape with only 4 houses km^−2^, fixation occurred in half the simulations, while the DEG became extinct with no lasting consequences in the remaining half. In 80% of the cases where the DEG became fixed, the population was driven to extinction; in the remaining 20% of these cases the population continued to persist through the course of the simulation although the population size was greatly reduced from the pre-DEG level.

### DEG dynamics are more variable in a seasonal environment

**Fig. 2:**
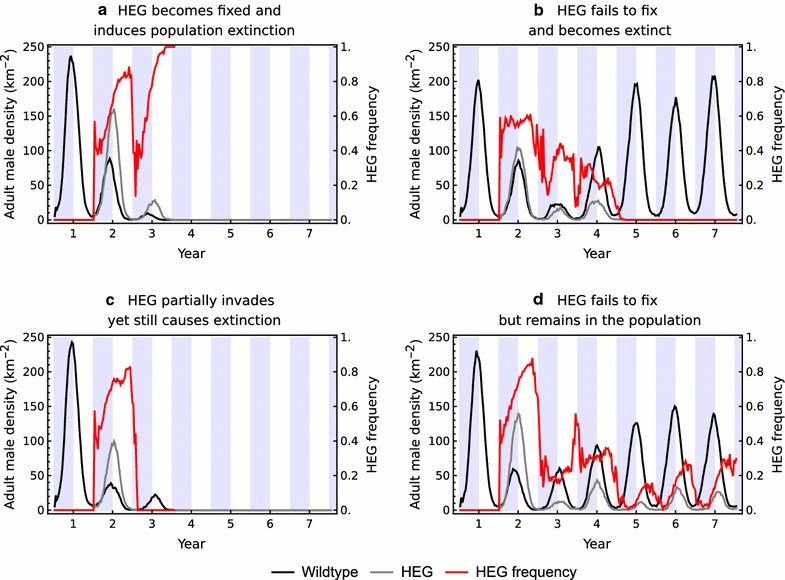
Examples of the simulated outcomes following a HEG release (blue shading indicates wet seasons) with average breeding site density 142 km^−2^, showing the densities of wildtype and HEG bearing adult males (black and grey lines respectively), and the corresponding HEG frequency (red). Demographic parameters follow North et al. [16]

In an environment with an *average* breeding site density of 142 km^−2^ (and the same house density of 4 km^−2^), yet with seasonal variation in the realized density at any time, a wider range of dynamics are possible (Fig. 2). Now, fixation occurred in 34% of simulations and invariably led to population extinction (Fig. 2a). The DEG became extinct with no lasting consequence to the population in 49% of simulations (Fig. 2b). In the remaining simulations, the DEG induced extinction despite first becoming lost from the population (17% of simulations, Fig. 2c) or, occasionally, the DEG and wildtype males both remained in the population throughout the simulated period of 6 years post release (0.4%, Fig. 2d). A common feature of these dynamics is a tendency for the DEG frequency to exhibit roughly annual fluctuations (red lines in Fig. 2). If a DEG becomes fixed in a wet season, the population is likely to become extinct in the following dry season because the scarcity of breeding sites results in low population densities, where stochastic effects frequently lead to extinction. If a DEG fails to fix in a given wet season, it may become lost from the population in the following dry season. In most simulations, the population will recover after the DEG is lost, although this may take several years. In some simulations, however, the remaining population is so reduced in numbers due to the effects of the invasion, that it is sensitive to stochastic events and it too becomes extinct during a subsequent dry season.

### The start of the wet season is the optimal time for release

**Fig. 3:**
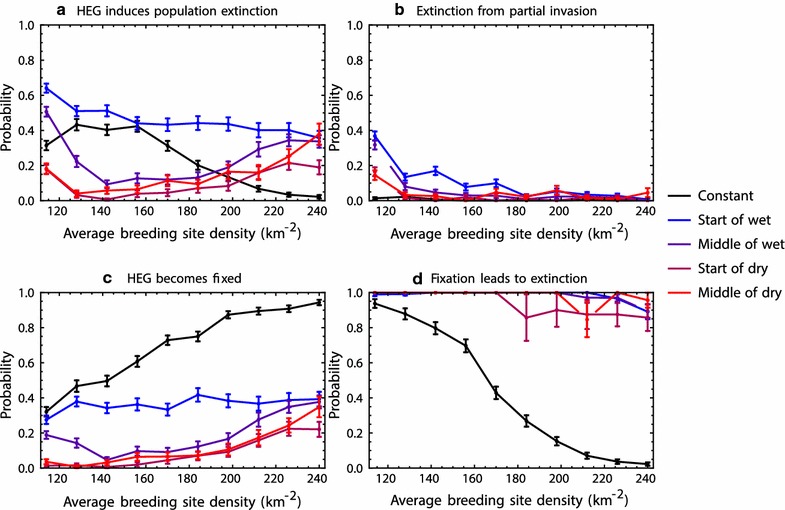
The impact of release timing (indicated by line colour) in a seasonal environment, and in a constant environment (black), as a function of average breeding site density. **a** The unconditional probability that the HEG causes population extinction, which is decomposed (a = b + c × d) into (**b**) the probability that a HEG that does not spread to fixation yet still induces a population extinction, and the probability that a fixed HEG induces extinction (c × d). The error bars indicate 95% confidence intervals based on a normal approximation with c.200 simulation runs. In these simulations, female numbers range from  ≈  6000 to 15,000 in the rainy season depending on the value of *θ*_*B*_ (the number of juveniles is about ×10 these quantities). Demographic parameters are the same as in Fig. 2

The relative probabilities of DEG loss and population suppression are influenced by both the time of year of the DEG release, and by the average density of breeding sites (Fig. 3). Extinction is most likely if the DEG is released at the start of the wet season (blue lines in Fig. 3) and least likely if the release is at the start of the dry season (magenta lines).

**Fig. 4:**
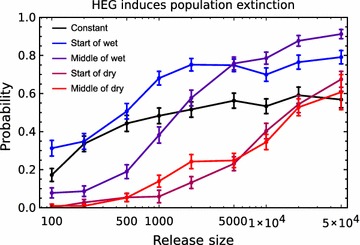
The effect of release size (the number of HEG bearing males introduced) on the probability of population extinction. The error bars indicate 95% confidence intervals based on a normal approximation with c.200 simulation runs. The parameters and colouration follow Fig. 3, with average breeding site density = 128 km^−2^

Whilst, in favourable conditions, a DEG is expected to increase to fixation due to its biased inheritance, in small populations a DEG may become extinct due to the released males failing to mate. The probability that a DEG overcomes stochastic loss to become fixed is therefore dependent on the size and growth rate of the wild-type population. In the first few weeks after a release, the risk of stochastic loss is highest if the release occurs in the dry season and lowest if it occurs in the wet season, because unmated females are least and most abundant at these times respectively. Over a longer period, the risk of stochastic loss is lowest if the release occurs at the start of the wet season, because this allows a longer period of favourable conditions (i.e. a relative abundance of unmated females) for the DEG to become established in the population (Fig. 3c). An additional benefit of releasing at this time is that for a given release size the initial DEG frequency is relatively high because the target population is small. If the initial frequency is high, fixation may occur during the first wet season leading to population extinction in the following dry season. This dependence on initial frequency means that the release size is generally more important in seasonal than constant environments, and large releases may compensate for sub-optimal release timing (Fig. 4).

### In seasonal environments, extinction is easier to achieve if the average breeding site density is low or high rather than intermediate

Irrespective of release time, the DEG was found to be more likely to induce extinction in seasonal environments with a low rather than an intermediate average breeding site density, in contrast to the trend when conditions are constant (Fig. 3a). This partly reflects a higher initial DEG frequency in the case of low average breeding site density, because the target population is smaller, and thus a greater chance of fixation in the year of release. In addition, the possibility of extinction occurring after the DEG is lost from the population is more likely if the average breeding site density is low (Fig. 3b). If breeding sites are generally rare, the mosquito intrinsic population growth rate will be particularly small during the dry season; in these conditions a population that has been suppressed by the impact of the DEG may become extinct due to stochastic effects.

In contrast, it was found that the probability of extinction increases with breeding site density in environments above a threshold, relatively high average density. A higher density of breeding sites reduces the risk of a DEG becoming stochastically lost from the target population, and thus promotes DEG establishment.

These results suggest that the role of initial DEG frequency is more important when breeding sites are generally rare, while DEG loss in the dry season is important when breeding sites are more abundant. However, the balance of these factors depends on release timing, and it is expected that detailed models will be required to understand their relative importance in specific scenarios.

### Random variation in rainfall generally increases the probability that a DEG will induce population extinction

**Fig. 5:**
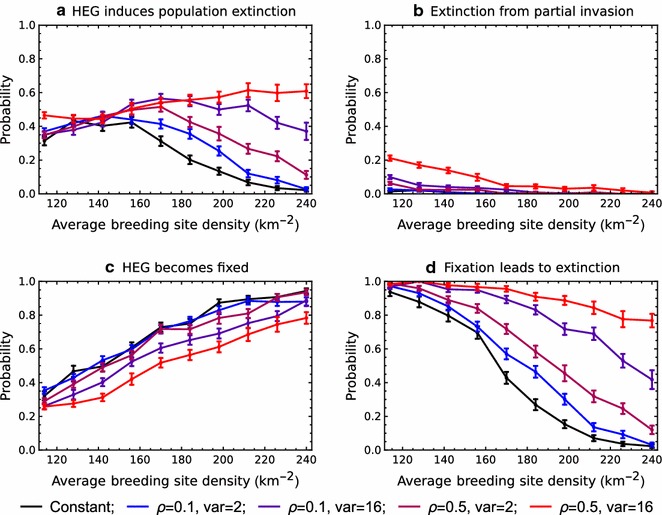
The impact of HEG releases in environments with stochastic variability in rainfall (coloured lines), and in a constant environment (black), as a function of average breeding site density. The simulated rainfall series were constructed with low (blue and purple) or high (magenta and red) levels of autocorrelation, and with low (blue and magenta) or high (purple and red) levels of variance. The error bars indicate 95% confidence intervals based on a normal approximation with c.200 simulation runs. Demographic parameters are the same as in Fig. 2

Next, the role of random weather variation is investigated, by allowing variance in the distribution of daily rainfall (controlled by *σ*) and autocorrelation in the rainfall time-series (controlled by *ρ*; Fig. 5). In cases of low variance and low autocorrelation, it was observed that there were only minor effects of variability on the outcome of a DEG release (compare blue versus black lines in Fig. 5a). The probability that a DEG induces population extinction increases, however, if either the variance in rainfall is increased (compare the magenta versus blue lines) or if the rainfall time-series becomes more autocorrelated (compare the purple versus blue lines). It was found that the probability of extinction is highest if the rainfall distribution has both high variance and high autocorrelation (the red line in Fig. 5a).

Random variability in rainfall increases the probability of extinction because it increases the variability in the dynamics of the target mosquito population. If a DEG becomes fixed in a variable climate, the population is more likely to become extinct because of demographic stochasticity in a subsequent drought (Fig. 5d). Moreover, if the variability is great, the impact of a DEG may lead to population extinction even after the DEG becomes itself lost from the population (Fig. 5b) for the reasons given above. These effects of variability are to some extent lessened by a reduced probability of fixation (Fig. 5c). Rainfall variability makes DEG establishment more difficult since a release may occur during drought, when there are few available breeding sites and stochastic loss of a rare allele is more likely. For the parameter ranges investigated, it was found that the greater potential impact of a DEG in a variable environment almost always outweighed the greater difficulty in establishment.

Note that this analysis assumes the releases occur on random dates unrelated to weather conditions, whereas an actual release programme may have some capacity to delay a release if conditions are particularly adverse. A more systematic strategy to dealing with an unpredictable climate may be to release multiple time-delayed batches of DEG-bearing mosquitoes rather than a single large batch.

### In models with more realistic rainfall, releases during the start of the wet season are normally (but not always) optimum

**Fig. 6:**
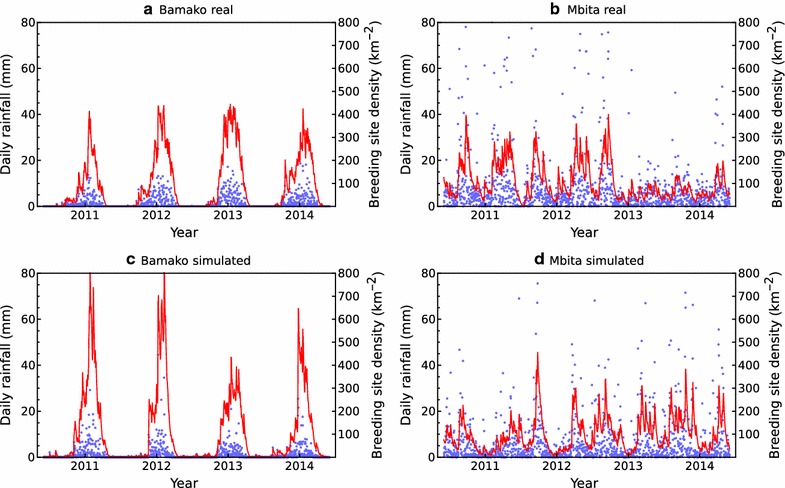
Rainfall data from Bamako, Mali and Mbita, Kenya (blue dots **a**, **c**), and corresponding time series simulated from the rainfall model with fitted parameters (**b**, **c**). The red lines plot the breeding site densities computed from the rainfall time series by the method outlined in the text, with *χ* = 0.1 and *κ* = 100

Now, the effect of release time on the probability of population extinction for sparse landscapes with low, medium, and high breeding site densities with realistic rainfall based on data from Bamako, Mali, and Mbita, Kenya is investigated. The rainfall model was fitted using 20 years of daily precipitation data for each site. The model produced rainfall patterns that visually resembled the observed data (Figs. 1, 6), while a more formal comparison [30] showed that the model replicated a range of important rainfall summary statistics (Table 1).

Simulations of mosquito populations in either of these locations typically end in population extinction during the dry season (Bamako) or a relative dry spell (Mbita), even in the absence of DEGs. The issue of how mosquito populations survive dry periods remains controversial, with aestivation and migration from permanent breeding sites the most likely explanations [31]. Neither of these mechanisms were present in the initial model, but prompted by the authors’ knowledge of these sites, the presence of some permanent water bodies was included in an extended model. Specifically, it was assumed that permanent breeding sites made up 35% of total sites in Bamako, and 20% in Mbita. These choices are somewhat arbitrary but represented the minimum permanent breeding site ratios that resulted in persistent wild-type populations in at least 99% of simulations.

**Fig. 7:**
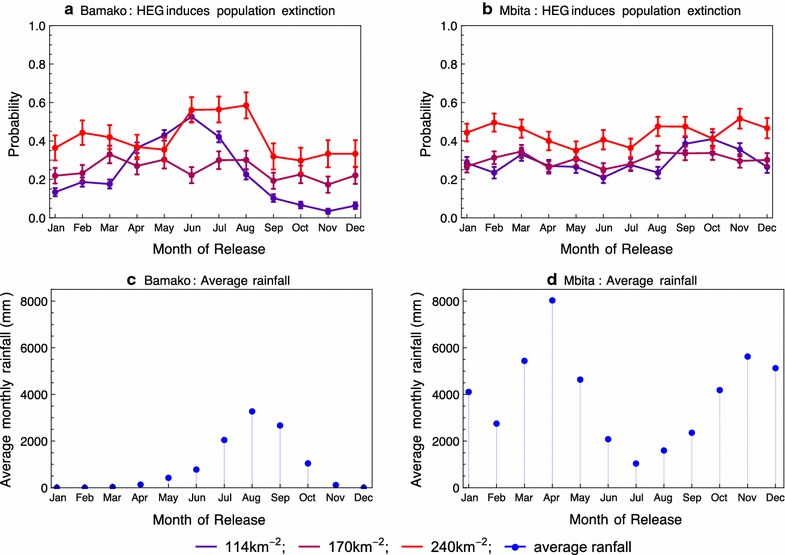
Simulated HEG releases in Bamako and Mbita (**a**, **b**). The probability of extinction depends on the average density of breeding sites in the environment (determined by line colouration) and the month of release. Graphs **c**, **d** show the average monthly rainfall over the 20 years of data we collected for each location. The error bars indicate 95% confidence intervals based on a normal approximation with c.200 simulation runs

In both locations, population extinction occurred more often in landscapes with a high rather than medium or low average density of breeding sites (compare red versus magenta and purple lines in Fig. 7). Release timing was most important in low density landscapes in the case of Bamako, where the probability of extinction markedly increases if the release occurs at the onset of the wet season. Seasonality at Mbita is less pronounced than at Bamako (compare Fig. 6a, b), and the effects of both breeding site density and release timing are weaker (Fig. 7b).

The probability of extinction increased with breeding site density because the risk of the stochastic loss of the DEG after introduction is lower in larger target populations with more unmated females for the introduced males to mate with. This corresponds with the earlier result in this study that the probability of fixation increases with average breeding site density across a wide range of parameters, albeit more strongly in constant than seasonal environments (Fig. 3c). Although this analysis of idealized seasonal environments suggested that fixation may lead to population suppression rather than extinction in landscapes where the average breeding site density is particularly high (Fig. 3d), this outcome was not observed in these simulations with more realistic rainfall patterns.

The more marked effect of release timing in the strongly seasonal environment of Bamako, and in landscapes with the lowest average breeding site density, is also in keeping with the analysis based on idealized seasonal environments (Fig. 3). In an environment with a severe dry season, releases at the start of the wet season minimize the risk of the DEG becoming lost in its first few weeks after introduction. Additionally, the vulnerability of wild-type populations in landscapes with low quality breeding sites means that extinction can occur even if a DEG only manages to spread to a portion of the landscape before its loss. This possibility means that judicious choice of release time, which increases the probability that a DEG survives its first few days after introduction, is even more important for low breeding site density landscapes.

## Discussion

Mosquito population suppression or extinction using DEGs or a similar technology may be very valuable as part of a malaria eradication campaign, especially in circumstances where existing tools are difficult to implement. Mathematical modelling has a key role in demonstrating the strategic feasibility of DEG-based approaches [7, 10, 14–18], but also in optimizing their tactical deployment in different environments. The aim of this paper has been to add temporal variation in mosquito breeding site density driven by fluctuating rainfall to our previous model of mosquito dynamics in a spatially heterogenous landscape, and then to explore the utility of DEGs in highly seasonal environments when introduced at different times of the year. The precipitation model that is introduced here is highly flexible, and a wide range of realistic rainfall patterns can be described using a small number of parameters. In Additional file 1, the authors have included computer code (written in the software *Mathematica*) for fitting the model to rainfall time-series data, which is hoped will be helpful for research in this area. The results in this paper suggest that DEG-based programmes can be successful in interrupting malaria transmission in a wide range of climates, although the timing of DEG release may be important in environments where rainfall is highly variable.

It was most often found that DEGs should be released at the start of the wet season when populations of the target species are growing at the fastest rate, since this maximizes the probability that the genetic construct will successfully establish and subsequently spread to fixation. Such a strategy is straightforward in seasonal environments with a predictable wet season, for example in the Sahelian environments typified by the rainfall patterns experienced at Bamako [32]. In environments where rainfall is less predictable, it may be harder to plan releases to coincide with wet periods, and here a bet-hedging strategy of ‘few and often’ releases is often preferable to larger releases at a particular time of year. In practice the chance of success will be enhanced using local knowledge, for example making releases near permanent breeding sites with a more stable target population. Although population fluctuations driven by rainfall will increase the difficulty of establishing a DEG, it may also heighten the impact of those DEGs that do establish. It was found that a DEG once established can be more likely to suppress a target population if rainfall is variable because of an increased probability of stochastic extinction during dry periods.

In the absence of data on the spatial density of breeding sites, a range of breeding site densities was investigated that influenced the establishment and spread of a DEG. These results demonstrate that the performance of a DEG may be affected by seasonality only for landscapes with relatively sparse mosquito resources. In contrast, for landscapes with more abundant resources the spread of a DEG is assured [16], meaning that seasonality may play a smaller role. However, real landscapes are likely composed of patches of higher and lower densities of mosquito resources. The lower density regions may affect the spread of a DEG in much the same way that a road limits the spread of a forest fire. Therefore, the performance of a DEG in these types of landscape was investigated. It was found that mosquitoes were unable to persist in some of the highly seasonal environments which were modelled, for example those with rainfall patterns similar to Bamako. This finding quantifies the intuition of many mosquito biologists who have highlighted the difficulty of explaining how mosquito populations survive dry seasons that last significantly longer than an individual’s expected lifespan. For example, *Anopheles* mosquitoes are extremely hard to find during a Sahelian dry season [31, 33, 34], but they invariably become common soon after the wet season begins. In some places, mosquitoes will continue to breed in water bodies that persist through the dry season (the assumption introduced into the model), but in their absence, there are two main hypotheses to explain this seeming paradox. The first is that adult mosquitoes undergo a dry season diapause (aestivation) by hiding in (currently unknown) shelters. The second contends that large numbers of adults migrate, possibly over large distances, soon after the onset of each wet season to repopulate suitable habitat [31, 35, 36]. The former hypothesis is supported by mark-recapture observations: remarkably, Lehmann et al. [35] discovered a female *Anopheles gambiae* s.s. still alive over 7 months after being marked in the previous wet season. There is also some experimental evidence of aestivation [36], while circumstantial evidence is provided by the rapidity of the population increase at the beginning of the wet season [35]. The migration hypothesis is supported by population survey data (some populations seem to disappear without trace during the dry season and return only after some delay during the following wet season, [31]). It is possible that different members of the *Anopheles gambiae* s.l. complex use different strategies; recent studies in Mali have suggested that *Anopheles coluzzii* aestivates while the closely related *An. gambiae* s.s. persists by long distance migration [31, 36].

Understanding exactly how mosquito populations bridge dry seasons is important and could affect DEG deployment strategies. It is very hard to find mosquitoes during the dry season and hence it is likely that if aestivation occurs that only a relatively small fraction of the population is involved. The probability of stochastic extinction during the dry season may thus be higher than in the model simulations reinforcing the advantage of allowing time for the DEG to spread during the previous wet season. If dry season survival is due to long distance migration, then the local population will be ‘replaced’ each year and a DEG that establishes in one season will be unlikely to persist. In this case, it will be critical to identify the source population or populations and to make releases in their vicinity. Provided these populations can be found, mosquito migration is likely to facilitate DEG spread and increase the likelihood of population suppression or extinction.

There have been studies of other possible mosquito control strategies that involve introducing modified insects at different times of year. *Wolbachia* are symbiotic bacteria that are found in many insect species, including mosquitoes, and spread through populations primarily via a mechanism known as cytoplasmic incompatibility [37]. *Wolbachia* interferes with arbovirus transmission and its introduction is currently being explored as a way of controlling dengue fever [38, 39], which is vectored primarily by *Aedes aegypti* mosquitoes. The success of a *Wolbachia* introduction depends on whether the frequency of infected mosquitoes after a release exceeds a threshold, and thus there is a strong advantage to making releases at the time of minimum mosquito density, typically the height of the dry season [40]. Genetic methods based on SIT (sterile insect technique) are also being explored as a method of suppressing *Aedes aegypti* [41]. The impact of an SIT programme depends on the ratio of released to wildtype males and so releases at the time of lowest population density is again optimum.

In addition to the better understanding of dry season population dynamics highlighted above, there are other ways in which tactical models of DEG implementation might be improved. The models that were used here assume a heterogeneous landscape specified by the variances and covariance in the spatial distribution of larval breeding and adult feeding sites. When potential release sites are identified these arbitrary landscapes should be replaced by the actual distribution of breeding and feeding sites, possibly estimated using GIS and related technologies, combined with local meteorological data. Field studies that improve understanding of the link between rainfall and breeding habitat will also be extremely helpful. The current model assumes that rainfall always leads to an increase in mosquito population size but in some circumstances heavy rainfall will wash away larvae from their breeding sites [42]. It would also be valuable to include other biotic variables such as temperature, which has a nonlinear effect on survival [43], modulates the rate of juvenile development [24], and affects the duration of the gonotrophic cycle [25]. Since there exist regions with strong seasonal variation in rainfall and temperature, changes in these variables may combine to affect significantly population dynamics [43]. Indeed, it has been suggested that partly because of its effect on vector population dynamics, temperature is correlated with malaria prevalence [42, 44]. It will also be important to incorporate more realistic male movement and swarming behaviour in the model [45] as this is likely to both increase the outcrossing rate and reduce competition among transgenic siblings, both of which should increase the spread, persistence, and efficacy of the driving Y.

DEGs can be used in a variety of different ways [14] in addition to causing sex ratio bias through a driving Y chromosome, as modelled here. The modelling framework can be extended to explore the use of DEGs to “knock out” genes essential for survival or fecundity, or to “knock in” a beneficial gene, for example one coding for a product that interrupts malaria transmission. Resistance to a DEG may arise via a mutation at the target site or via a trans-acting mutation at a different locus that suppresses the expression of the nuclease [46]. It may also arise from selection of a resistant allele originally present in the standing genetic variation [47]. The probability of target site resistance can be reduced by designing DEGs that cut multiple adjacent sites simultaneously [48, 49] or, in the case of a Y-drive DEG, by targeting a site that is repeated on the X-chromosome but absent elsewhere [12]. Beaghton et al. [50] investigated the likelihood that resistance will evolve against Y-drive using a model of a single well mixed population which may be subject to seasonality (modelled sinusoidally). They found that seasonality reduces the probability of a resistant allele establishing for a Y-drive transgene, to a degree that depends on the timing of the Y-drive release (dry season releases had the lowest probability of resistance arising, [50]). Resistant alleles are less likely to arise in low-density populations, as considered in this paper, than in large well mixed populations [47, 50]. It is less clear how random variability in rainfall will influence the establishment of resistant alleles, and the modelling framework that is presented here may help answer this question.

## Conclusion

It has been demonstrated that mosquito population control using a driving homing endonuclease gene (DEG) that causes severe sex ratio distortion can be a viable strategy in a highly seasonal environment, and that the precise timing of releases during the year can be important in maximizing success. The authors finish by noting that though the use of DEGs and other gene-editing strategies is a novel technology with great promise, it is essential that a rigorous governance framework is set up to regulate its use, both to avoid any negative health or environmental impacts and to assure a licence to operate by civil society in the countries where it may be employed.

## Additional files

**Additional file 1.** RainDataB contains the daily precipitation data for Bamako, Mali and Mbita, Kenya, obtained from European Centre for Medium-Range Weather Forecasts [29].

**Additional file 2.** Rainfall model fitter. A *Mathematica* file which fits the rainfall model paper to precipitation data.

## References

[1] WHO (2014). World malaria report 2014.

[2] WHO (2015). Global technical strategy for malaria 2016–2030.

[3] Le Menach A, Takala S, McKenzie FE, Perisse A, Harris A, Flahault A (2007). An elaborated feeding cycle model for reductions in vectorial capacity of night-biting mosquitoes by insecticide-treated nets. Malar J.

[4] Ferguson HM, Dornhaus A, Beeche A, Borgemeister C, Gottlieb M, Mulla MS (2010). Ecology: a prerequisite for malaria elimination and eradication. PLoS Med.

[5] Read AF, Lynch PA, Thomas MB (2009). How to make evolution-proof insecticides for malaria control. PLoS Biol.

[6] Tanner M, Greenwood B, Whitty CJM, Ansah EK, Price RN, Dondorp AM (2015). Malaria eradication and elimination: views on how to translate a vision into reality. BMC Med.

[7] Burt A (2003). Site-specific selfish genes as tools for the control and genetic engineering of natural populations. Proc Biol Sci.

[8] Mueller JE, Bryk M, Loizos N, Belfort M (1993). 4 Homing endonucleases. Cold Spring Harbor Monograph Archive.

[9] Hammond A, Galizi R, Kyrou K, Simoni A, Siniscalchi C, Katsanos D (2016). A CRISPR-Cas9 gene drive system targeting female reproduction in the malaria mosquito vector *Anopheles gambiae*. Nat Biotechnol.

[10] North A, Burt A, Godfray HCJ (2017). How driving endonuclease genes can be used to combat pests and disease vectors. BMC Biol.

[11] Windbichler N, Menichelli M, Papathanos PA, Thyme SB, Hui L, Ulge UY (2011). A synthetic homing endonuclease-based gene drive system in the human malaria mosquito. Nature.

[12] Galizi R, Doyle LA, Menichelli M, Bernardini F, Deredec A, Burt A (2014). A synthetic sex ratio distortion system for the control of the human malaria mosquito. Nat Comm.

[13] Bernardini F, Galizi R, Menichelli M, Papathanos PA, Dritsou V, Marois E (2014). Site-specific genetic engineering of the *Anopheles gambiae* Y chromosome. Proc Natl Acad Sci USA.

[14] Deredec A, Burt A, Godfray HCJ (2008). The population genetics of using homing endonuclease genes in vector and pest management. Genetics.

[15] Deredec A, Godfray HCJ, Burt A (2011). Requirements for effective malaria control with homing endonuclease genes. Proc Natl Acad Sci USA.

[16] North A, Burt A, Godfray HCJ (2013). Modelling the spatial spread of a homing endonuclease gene in a mosquito population. J Appl Ecol.

[17] Eckhoff PA, Wenger EA, Godfray HCJ, Burt A (2017). Impact of mosquito gene drive on malaria elimination in a computational model with explicit spatial and temporal dynamics. Proc Natl Acad Sci USA.

[18] Beaghton A, Beaghton PJ, Burt A (2016). Gene drive through a landscape: reaction–diffusion models of population suppression and elimination by a sex ratio distorter. Theor Popul Biol.

[19] Gimnig JE, Ombok M, Kamau L, Hawley WA (2001). Characteristics of larval anopheline (Diptera: Culicidae) habitats in Western Kenya. J Med Entomol.

[20] Shililu J, Ghebremeskel T, Seulu F, Mengistu S, Fekadu H, Zerom M (2003). Larval habitat diversity and ecology of anopheline larvae in Eritrea. J Med Entomol.

[21] Fillinger U, Sonye G, Killeen GF, Knols BGJ, Becker N (2004). The practical importance of permanent and semipermanent habitats for controlling aquatic stages of *Anopheles gambiae* sensu lato mosquitoes: operational observations from a rural town in western Kenya. Trop Med Int Health.

[22] Koenraadt CJM, Githeko AK, Takken W (2004). The effects of rainfall and evapotranspiration on the temporal dynamics of *Anopheles gambiae* s.s. and *Anopheles arabiensis* in a Kenyan village. Acta Trop.

[23] Bárdossy A, Plate EJ (1992). Space-time model for daily rainfall using atmospheric circulation patterns. Water Resour Res.

[24] Bayoh MN, Lindsay SW (2003). Effect of temperature on the development of the aquatic stages of *Anopheles gambiae* sensu stricto (Diptera: Culicidae). Bull Entomol Res.

[25] Gillies MT (1953). The duration of the gonotrophic cycle in *Anopheles gambiae* and *Anopheles funestus*, with a note on the efficiency of hand catching. East Afr Med J.

[26] Mutuku FM, Alaii JA, Bayoh MN, Gimnig JE, Vulule JM, Walker ED (2006). Distribution, description, and local knowledge of larval habitats of *Anopheles gambiae* s.l. in a village in western Kenya. Am J Trop Med Hyg.

[27] Fillinger U, Lindsay SW (2006). Suppression of exposure to malaria vectors by an order of magnitude using microbial larvicides in rural Kenya. Trop Med Int Health.

[28] Nmor JC, Sunahara T, Goto K, Futami K, Sonye G, Akweywa P (2013). Topographic models for predicting malaria vector breeding habitats: potential tools for vector control managers. Parasit Vectors.

[29] Dee DP, Uppala SM, Simmons AJ, Berrisford P, Poli P, Kobayashi S (2011). The ERA-interim reanalysis: configuration and performance of the data assimilation system. Q J R Meteor Soc.

[30] Bárdossy A, Stehlik J, Caspary HJ (2001). Generating of areal precipitation series in the upper Neckar catchment. Phys Chem Earth PT B.

[31] Dao A, Yaro AS, Diallo M, Timbiné S, Huestis DL, Kassogué Y (2014). Signatures of aestivation and migration in Sahelian malaria mosquito populations. Nature.

[32] Xue Y, Hutjes RWA, Harding RJ, Claussen M, Prince SD, Lebel T, Kabat P, Claussen M, Dirmeyer PA, Gash JHC, de Guenni LB, Meybeck M (2004). The Sahelian climate. Vegetation, water, humans and the climate. Global change—the IGBP series.

[33] Touré YT, Dolo G, Petrarca V, Traoré SF, Dao A, Carnahan J (1998). Mark–release–recapture experiments with *Anopheles gambiae* s.l. in Banambani Village, Mali, to determine population size and structure. Med Veter Entomol.

[34] Taylor C, Touré YT, Carnahan J, Norris DE, Dolo G, Traoré SF (2001). Gene flow among populations of the malaria vector, *Anopheles gambiae*, in Mali, West Africa. Genetics.

[35] Lehmann T, Dao A, Yaro AS, Adamou A, Kassogue Y, Diallo M (2010). Aestivation of the African malaria mosquito, *Anopheles gambiae* in the Sahel. Am J Trop Med Hyg.

[36] Adamou A, Dao A, Timbine S, Kassogué Y, Yaro AS, Diallo M (2011). The contribution of aestivating mosquitoes to the persistence of *Anopheles gambiae* in the Sahel. Malar J.

[37] Yen JH, Barr AR (1971). New hypothesis of the cause of cytoplasmic incompatibility in *Culex pipiens* L. Nature.

[38] Moreira LA, Iturbe-Ormaetxe I, Jeffery JA, Lu G, Pyke AT, Hedges LM (2009). A Wolbachia symbiont in *Aedes aegypti* limits infection with dengue, Chikungunya, and *Plasmodium*. Cell.

[39] Bian G, Xu Y, Lu P, Xie Y, Xi Z (2010). The endosymbiotic bacterium *Wolbachia* induces resistance to dengue virus in *Aedes aegypti*. PLoS Pathog.

[40] Hancock PA, Sinkins SP, Godfray HCJ (2011). Strategies for introducing *Wolbachia* to reduce transmission of mosquito-borne diseases. PLoS Negl Trop Dis.

[41] Thomas DD, Donnelly CA, Wood RJ, Alphey LS (2000). Insect population control using a dominant, repressible, lethal genetic system. Science.

[42] Reiner RC, Geary M, Atkinson PM, Smith DL, Gething PW (2015). Seasonality of *Plasmodium falciparum* transmission: a systematic review. Malar J.

[43] Craig MH, Snow RW, Le Sueur D (1999). A climate-based distribution model of malaria transmission in sub-Saharan Africa. Parasitol Today.

[44] Gething PW, Patil AP, Smith DL, Guerra CA, Elyazar IR (2011). A new world malaria map: *Plasmodium falciparum* endemicity in 2010. Malar J.

[45] Diabaté A, Dao A, Diallo M, Huestis DL, Lehmann T (2011). Spatial distribution and male mating success of *Anopheles gambiae* swarms. BMC Evol Biol.

[46] Weterings E, Chen DJ (2008). The endless tale of non-homologous end-joining. Cell Res.

[47] Unckless RL, Clark AG, Messer PW (2017). Evolution of resistance against CRISPR/Cas9 gene drive. Genetics.

[48] Esvelt KM, Smidler AL, Catteruccia F, Church GM (2014). Concerning RNA-guided gene drives for the alteration of wild populations. Elife.

[49] Alphey L (2016). Can CRISPR-Cas9 gene drives curb malaria?. Nat Biotechnol.

[50] Beaghton A, Hammond A, Nolan T, Crisanti A, Godfray HCJ, Burt A (2017). Requirements for driving antipathogen effector genes into populations of disease vectors by homing. Genetics.

